# A Vanishing Cecal Mass: A Rare Gastrointestinal Manifestation of Systemic Mastocytosis

**DOI:** 10.7759/cureus.20784

**Published:** 2021-12-28

**Authors:** Krista Newman, Gregory Vercellotti, Dale Snover, Timothy Peterson, Eugenia Shmidt

**Affiliations:** 1 Gastroenterology, Hepatology and Nutrition, University of Minnesota, Minneapolis, USA; 2 Hematology, Oncology and Transplantation, University of Minnesota, Minneapolis, USA; 3 Laboratory Medicine and Pathology, University of Minnesota, Minneapolis, USA; 4 General Surgery, Meeker Memorial Clinic, Litchfield, USA

**Keywords:** endoscopy, colonoscopy, mast cell, systemic mastocytosis, colon mass

## Abstract

Systemic mastocytosis (SM) is a heterogeneous disease that often involves the gastrointestinal (GI) tract. Activation and accumulation of mast cells in GI organs can result in symptoms of abdominal pain, nausea and diarrhea along with organ damage with more aggressive disease. Mast cell degranulation can also result in anaphylactic reactions, which can be life-threatening. Recognition of GI manifestations is important for gastroenterologists to aid in diagnosis and management when GI involvement is suspected. Edema, small nodules, urticarial lesions and occasionally ulceration in the small bowel and colon are the most commonly described endoscopic findings. Here we describe a case of SM presenting as a large colonic mass and provide a brief review of the literature on GI involvement of SM.

## Introduction

Mast cells play sentinel roles in the modulation of the adaptive immune system and are found in mucosal and epithelial tissues throughout the body. Once activated, mast cells release potent mediators such as histamine, cytokines, proteases and chemokines. Pathologic accumulation and activation of mast cells can manifest with profound end-organ effects and debilitating symptoms with mast cell disease [1].

Systemic mastocytosis (SM) is a rare disease that is associated with a gain-of-function point mutation (D816V) in the KIT gene [2]. KIT encodes the transmembrane receptor protein kit (CD117) that is implicated in mast cell growth, division and survival. Mutations in KIT result in the overproduction of aberrant mast cells that accumulate in organs [3]. Gastrointestinal (GI) manifestations such as nausea, vomiting and abdominal pain are observed in up to 60%-80% of patients [4,5]. Mast cell degranulation can also result in life-threatening anaphylactic reactions; Brockow et al. reported that adults with SM have a 20%-50% risk for anaphylaxis [6]. Symptom presentation and diagnosis of SM can be challenging and is important for medical providers to recognize SM to avoid morbidity. Here, we describe an unusual case of SM that manifested as a colon mass on routine screening colonoscopy.

This article was previously presented as an abstract at the 2019 American College of Gastroenterology meeting on Sunday, October 27, 2019.

## Case presentation

**Figure 1:**
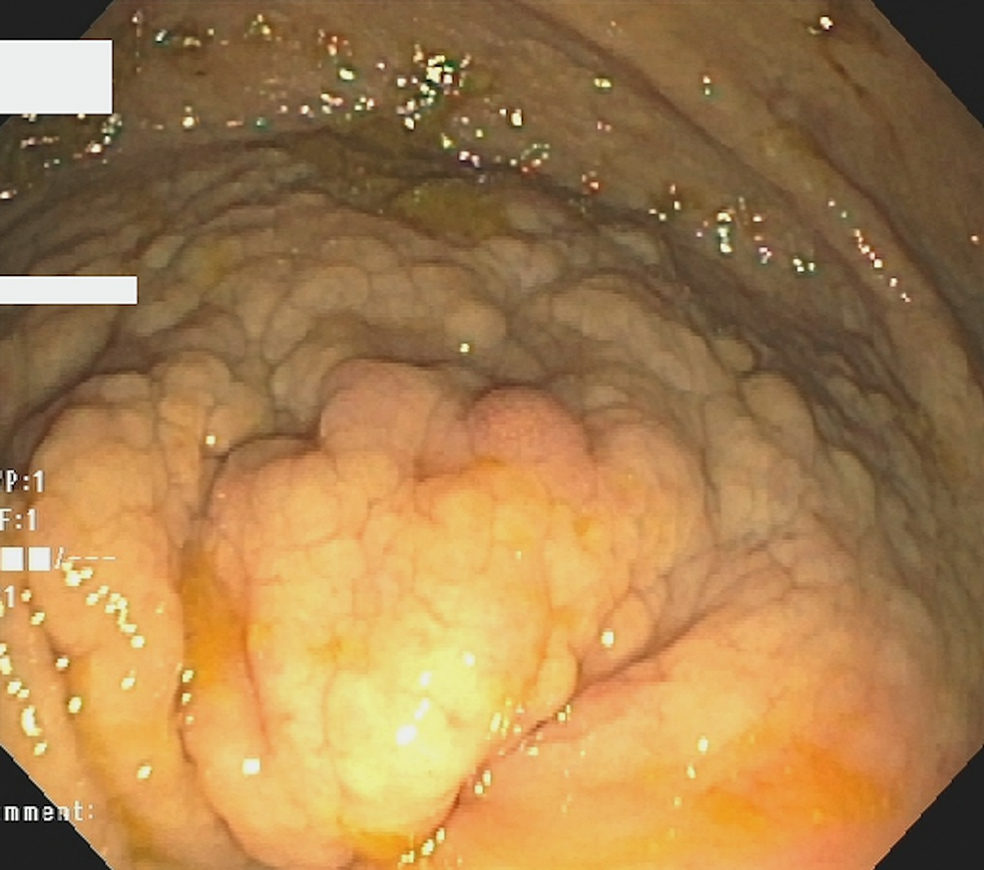
Eight by 12-centimeter polypoid mass involving the cecum and ileocecal valve

**Figure 2:**
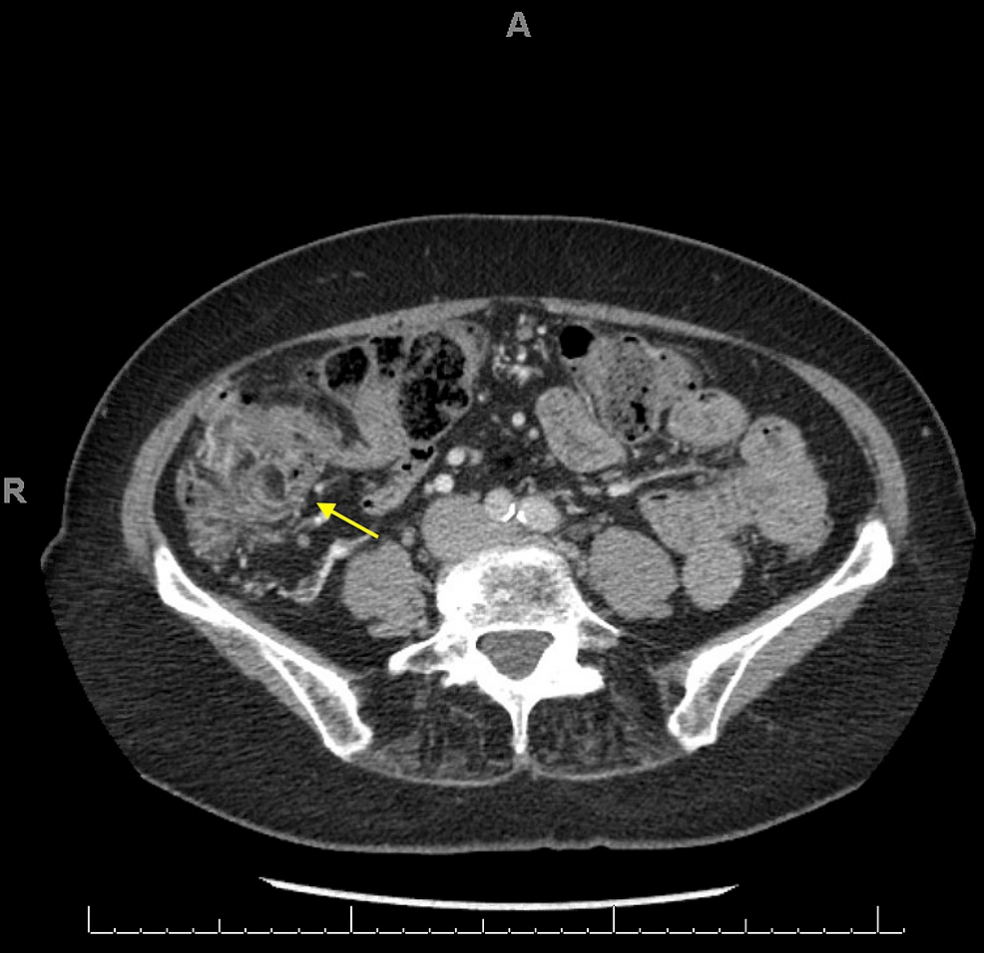
CT abdomen and pelvis showing mucosal thickening and hyperenhancement with slight vascular engorgement in the cecum and ascending colon

A 71-year-old asymptomatic female with urticaria pigmentosa was referred to a tertiary center gastroenterology clinic for evaluation of an 8cm by 12cm polypoid mass involving the cecum and ileocecal valve that was found on routine screening colonoscopy (Figure 1). Additional evaluation prior to our initial clinic visit included a CT scan that remarked on mucosal hyperenhancement with slight vascular engorgement and colonic wall thickening involving the cecum and lower portion of the ascending colon (Figure 2).

During our initial clinic visit, the patient reported a history of a diffuse skin rash that was present since her 20s, she was later diagnosed with urticaria pigmentosa in her 50s after she developed symptoms of pruritus. She had a thorough evaluation with dermatology including bone marrow biopsy, 24-hour urine testing, blood work and full body x-rays to assess for SM, which were negative at that time. She had been managed with cetirizine for symptoms of pruritus. She did not endorse any significant GI symptoms other than occasional diarrhea, nausea and vomiting only after consuming coffee or chocolate.

Investigations

Following the initial clinic visit, blood work was obtained and showed an entirely normal CBC with differential and comprehensive metabolic panel but was notable for an elevated serum tryptase level of 37.4 µg/L. The elevated serum tryptase level was suggestive of SM. We proceeded with a repeat colonoscopy to re-evaluate the mass along with an upper endoscopy because of the patient’s symptoms of nausea with the consumption of coffee or chocolate.

**Figure 3:**
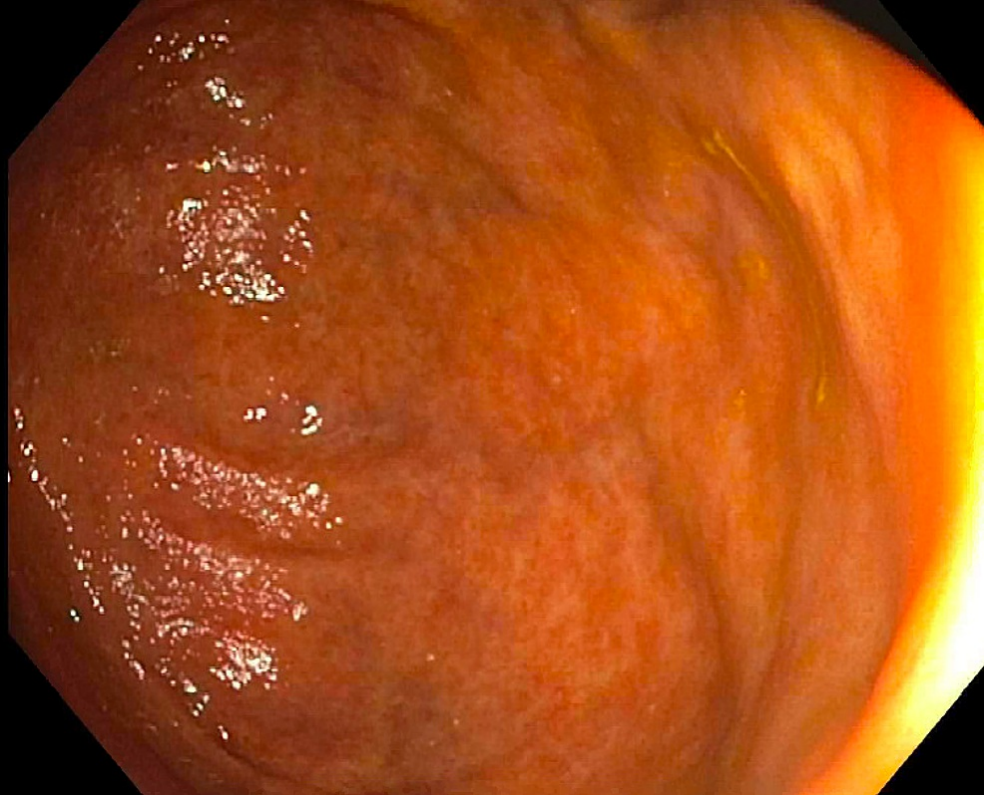
Colonoscopy with evidence of mildly erythematous mucosa and in the cecum with no obvious mass

**Figure 4:**
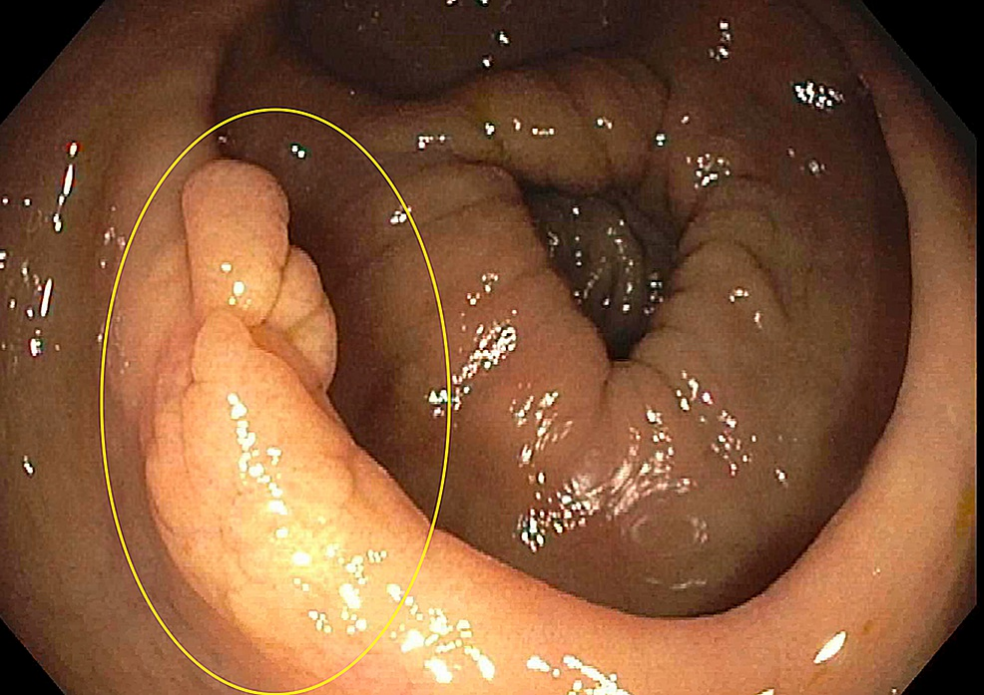
Slightly nodular mucosa in the transverse colon

**Figure 5:**
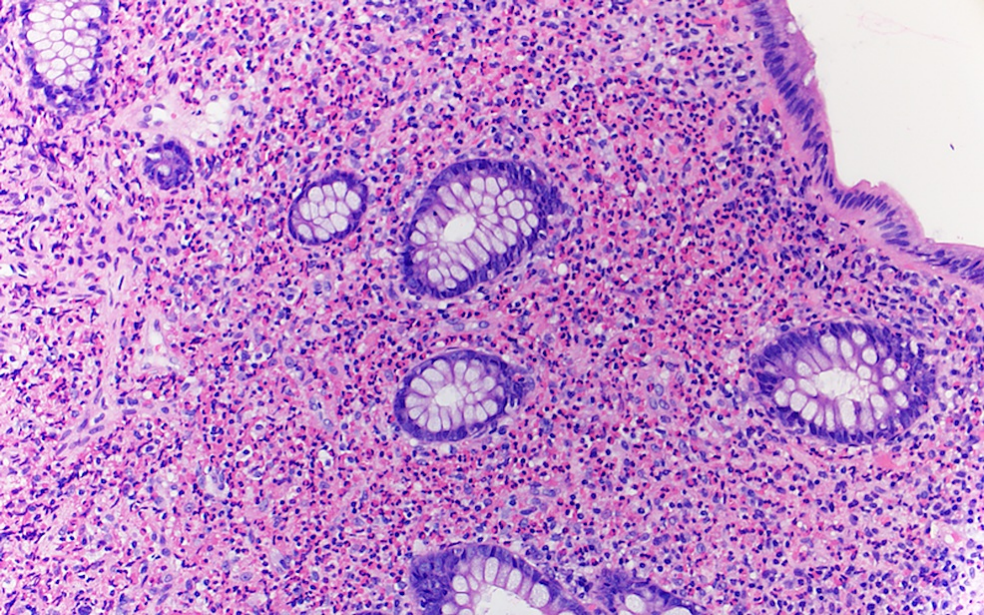
Hematoxylin and eosin staining of colonic mucosa with mixed mononuclear and eosinophilic infiltrate displacing normal crypts

**Figure 6:**
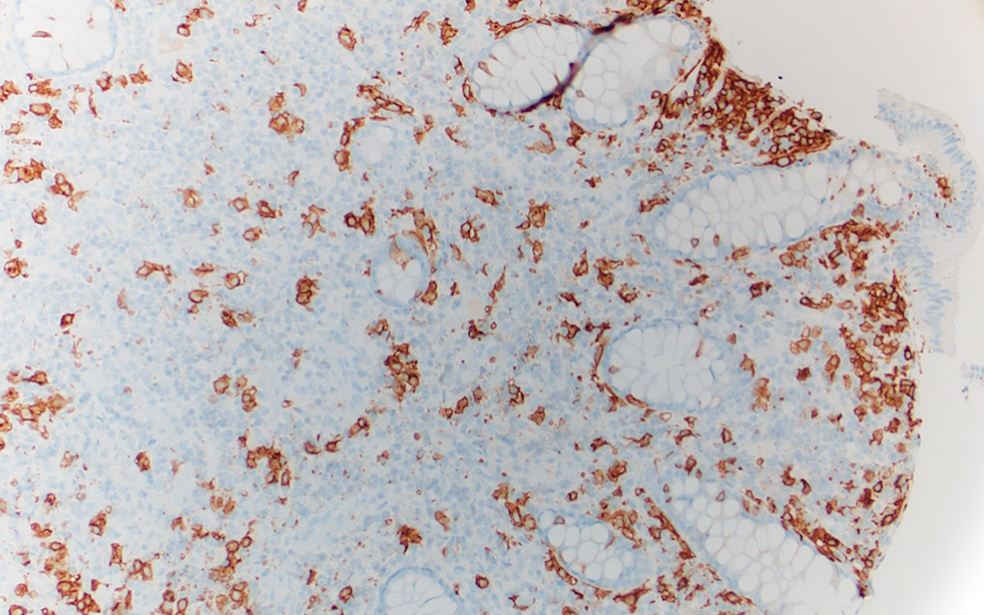
Colon biopsy with immunohistochemical staining for CD 117 confirming that mononuclear cells seen on hematoxylin and eosin are mast cells

The initial large, polypoid cecal mass was not present on repeat colonoscopy (Figure 3). In its place, there was mild erythema in the cecum and patchy, nodular appearing mucosa in the right colon (Figure 4). The upper endoscopy was notable only for mild, diffuse gastric erythema. Random and targeted biopsies of both the upper and lower GI tract were taken in the stomach, duodenum, ileum, cecum, ascending and transverse colon. Pathology from the biopsies from all sites revealed dense (>100 per high powered field) infiltration of mast cells that stained positive for CD117 and CD25 cell surface proteins and the presence of eosinophils in the lamina propria (Figures 4-6).

Differential diagnosis

Large colon masses may be related to a variety of causes including malignant lesions, lymphoma, infectious or inflammatory conditions. The urticarial/nodular nature of the colon mass with dense infiltration of mast cells on pathology in the context of a patient with urticaria pigmentosa was highly suggestive of mast cell disorder.

Although mast cell infiltration can be seen in other GI diseases, studies have shown that mast cells that co-express CD25 are specific for SM [7]. Thus, the presence of CD25 has aided in the diagnosis of SM. In 2001, the World Health Organization (WHO) proposed specific diagnostic criteria for SM with the most recent iteration released in 2016. The WHO criteria involve major and minor features that include the following: histologic evidence of dense mast cell infiltration in bone marrow or extracutaneous organs (major criterion), KIT point mutations (minor), mast cell cytomorphologic changes (minor), mast cell expression of CD25 and/or CD2 (minor) and elevated serum tryptase levels >20 ng/mL (minor). The diagnosis of SM can be made when either the major criterion with one minor criterion or three minor criteria are met.

In addition to diagnostic criteria to help diagnose SM the WHO have also described five subtypes of SM that range in severity from a slowly progressive, indolent process to a disease resulting in organ impairment and failure with associated reduced life expectancy. The five subtypes include indolent SM, smoldering SM, SM with associated hematologic neoplasm, aggressive SM and mast cell leukemia [7]. Our patient’s presentation was consistent with indolent SM with the multifocal involvement of mast cells with expression of CD25 in the GI tract along with an elevated serum tryptase level and otherwise no evidence of associated hematologic neoplasm, significant bone marrow involvement or other organ dysfunction.

Treatment

Treatment of patients with indolent SM involves a multi-disciplinary approach to address the following: education to avoid triggers, management of symptoms related to mast cell mediator release, evaluation and management of allergies and anaphylaxis, evaluation for Hymenoptera allergy, assistance with peri-operative management due to risk of mast cell degranulation and assessment for bone disease [8].

Following a diagnosis of SM, we coordinated a multidisciplinary clinic visit with colleagues in hematology with expertise in SM and carefully reviewed symptoms and potential triggers. Interestingly, despite dense infiltration of mast cells in the upper and lower GI tract the patient experienced only very subtle GI symptoms which included mild nausea and occasional diarrhea after consuming alcohol or chocolate. Our hematology colleagues also remarked on the absence of dermatographia which suggested minimal cutaneous mast cell mediator release, which was not an anticipated finding given the dense, multifocal mast cell involvement throughout the GI tract. First-line management of pruritus and GI symptoms includes the use of H1 and H2-receptor antagonists, respectively [8,9]. In our patient, cetirizine was continued with the addition of famotidine for occasional symptoms of heartburn and nausea with trigger foods. Most importantly, an epinephrine pen was prescribed due to the risk for anaphylactic reactions, even in the absence of prior history, given significant risk in this patient population.

Outcome and follow-up

Case series have found that patients with indolent SM have a normal life expectancy. However, a small subset may experience disease progression, which can be predicted by KIT mutations in all hematopoietic lineages [3,10]. Patients should have a periodic follow-up in the clinic to monitor for evidence of disease progression. It is also important to follow up on medical management and symptom response. A validated mastocytosis symptom assessment form found that fear of anaphylaxis and fatigue have the highest impact on patients’ quality of life [11,12].

Our patient continues to follow with hematology and is doing well in a three-year follow-up from the initial presentation to our clinic. Occasional symptoms of nausea or diarrhea with trigger foods continue to be well managed with famotidine and there has been no evidence of disease progression.

## Discussion

This case represents a rare presentation and new diagnosis of SM from a colon mass on screening colonoscopy in a patient with a history of urticaria pigmentosa. Systemic involvement can be difficult to recognize. Our patient reported subtle GI symptoms during our initial clinic visit. It is important to have a high index of suspicion for SM in adults with urticaria pigmentosa who report any GI symptoms. Medical providers should also recognize that in patients with known SM, GI symptoms are frequent and should be addressed. One of the largest case-control studies found that up to 59% of patients with SM had disabling symptoms [13]. The most commonly reported symptoms are pruritus, abdominal pain, diarrhea, nausea and vomiting and are the result of mast cell mediator release [4,13]. Aggressive subtypes of SM can manifest with more serious findings related to organ infiltration and dysfunction and can include GI bleeding, peptic ulcer disease, liver disease including non-cirrhotic portal hypertension and malabsorption [14-18]. Endoscopic evaluation should be pursued in adults with GI symptoms, particularly those with urticaria pigmentosa. One study showed that almost all patients with adult-onset skin disease were found to have SM after further evaluation which requires biopsies to assess for mast cell infiltration based on WHO criteria [19]. Gastroenterologists should be familiar with the variety of endoscopic findings that may be found on the exam.

Endoscopic abnormalities in SM are most often identified in the colon followed by the terminal ileum and upper GI tract [5,20]. Endoscopic findings include mucosal nodularity, pseudopolyps, aphthous ulcers, erythema, edema and abnormal folds [13,20]. To the best of our knowledge, this is the first description of a transient colon mass lesion that led to the diagnosis of SM.

Although upper GI findings are less common in SM, peptic ulcer disease may be an underdiagnosed phenomenon [13]. Up to 40% of patients who have had upper endoscopy were found to have peptic ulcer disease [4]. Esophageal complaints are rare but there have been reports of esophagitis and even variceal bleeding related to portal hypertension as a result of mast cell infiltration [16]. Even in the absence of endoscopic findings, there can still be dense mast cell infiltration. One case series of 23 patients with SM reported that 63% of patients with normal exams were found to have mast cells on random biopsy [20]. Although there is no clear guidance, Doyle et al. recommend taking multiple random biopsies at different sites as well as any areas of endoscopic abnormalities and use of immunohistochemistry staining for CD117 as a screening tool. Random biopsies can aid detection of the sometimes subtle, multi-focal aggregates of mast cells on biopsies in patients who have skin or other organ involvement of mastocytosis [3]. Following endoscopic evaluation, the WHO criteria may be used to aid in the diagnosis of SM.

## Conclusions

SM is a rare disease that often involves the GI tract. This case provides an example of a new diagnosis of SM in a patient with a history of urticaria pigmentosa who presented with a colon mass on screening colonoscopy. Recognition of GI symptoms followed by endoscopic evaluation of SM with GI mucosal biopsies with staining for CD117 and CD25 cell surface proteins is prudent for gastroenterologists since diagnosis of SM is the first step to initiate therapy to avoid morbidity. Clinicians should utilize multidisciplinary care for ongoing management and it is important to recognize that all subtypes of SM patients are at increased risk of mast cell degranulation and anaphylaxis.
